# A generation at risk: the unseen consequences of Nepal’s nursing shortage on undergraduate nursing students clinical education

**DOI:** 10.1186/s12912-024-02578-5

**Published:** 2024-12-21

**Authors:** Animesh Ghimire, Mamata Neupane

**Affiliations:** 1 School of Nursing, Chitwan Medical College, Bharatpur-5, Kailashnagar, Chitwan, Nepal; 2 Sustainable Prosperity Initiative Nepal, Thulo Kharibot, Baneshwor-31, Bhimsengola, Kathmandu, Nepal; 3 School of Nursing and School of Public Health, Chitwan Medical College, Bharatpur-5, Kailashnagar, Chitwan, Nepal

**Keywords:** Nursing shortage, Clinical Education, Student Resilience, AI Mentorship, Global South, Compromised learning

## Abstract

**Background:**

The global nursing shortage has particularly severe consequences in resource-constrained countries like Nepal, where a significant outflow of healthcare professionals exacerbates the crisis. While the impact on patient care, workforce dynamics, and organizational challenges within healthcare settings have been extensively researched, the profound implications of this crisis for nursing education remain underexplored. This qualitative study sheds light on the often-unseen consequences of the nursing shortage on undergraduate nursing students’ clinical education.

**Methods:**

Data were collected from three focus group discussions involving eighteen final-year nursing students from two tertiary institutions. The first two discussions took place in June 2024, with the third one in July 2024. Thematic analysis was employed to identify and interpret the key themes.

**Results:**

Four overarching themes emerged: (1) Compromised Learning, highlighting the detrimental impact on skill acquisition and student preparedness; (2) Abandoned in the Field, revealing the emotional toll of isolation, with students describing themselves as “ghosts, silently observing from the sidelines”; (3) Forging Ahead, showcasing the unintended resilience and resourcefulness of students seeking alternative knowledge sources; and (4) Digital Mentors, spotlighting the innovative use of Artificial Intelligence (AI) as a supplementary learning tool in overburdened clinical settings.

**Conclusion:**

The nursing shortage in Nepal has created a challenging and often isolating clinical learning environment (CLE) for students. While their resilience and adaptability are commendable, these qualities are not substitutes for nurse staffing shortages and inadequate mentorship. This study underscores the urgent need for systemic change, including increased investment in the nursing workforce, cultivating a supportive learning environment, and integrating technology into nursing education. These findings have implications for Nepal and other resource-constrained settings grappling with the challenges of a nursing shortage.

**Supplementary Information:**

The online version contains supplementary material available at 10.1186/s12912-024-02578-5.

## Background

Undergraduate nursing education is founded on integrating two key elements: the theoretical foundation and practical clinical training [1]. Within the clinical setting, students acquire, develop, and refine practical skills, linking them to the learned theoretical knowledge to address complex patient needs and deliver safe care. Nursing education’s inherent skill-based and technical nature necessitates substantial practical training for students [2]. Consequently, the clinical learning environment (CLE) plays a pivotal role in shaping the quality of nursing education and significantly influences students’ learning outcomes [3]. Clinical placements within the CLE are a mandatory and indispensable component of undergraduate nursing education, providing essential opportunities for students to develop the knowledge, skills, and professional identity necessary for competent practice [1]. These placements provide immersive, hands-on learning opportunities within dynamic healthcare settings, encompassing interactions with nurses, doctors, allied health staff, patients, and their families, all within the context of constantly shifting schedules and the complexities of patient care [4–6]. Clinical learning, therefore, is an indispensable component of the nursing curriculum. It enables students to apply theoretical knowledge, cultivate essential skills, and develop the competence necessary for professional nursing practice.

Nepal’s nursing education system comprises a bachelor’s degree program and a Proficiency Certificate Level in Nursing (PCL Nursing) Program designed to cultivate a mid-level technical nursing workforce [7]. The bachelor’s degree program caters to students who have completed Year 12 of secondary education and culminates in a Bachelor of Science in Nursing (BSc Nursing) degree. This four-year program provides comprehensive training, preparing graduates for diverse roles within the nursing profession [7]. The second pathway is designed for students who have completed Year 10 of secondary education and obtained a Proficiency Certificate Level in Nursing (PCL Nursing). This pathway leads to a Bachelor of Nursing Science (BNS) degree, a three-year program [7]. Both BSc Nursing and BNS graduates play a crucial role in fulfilling Nepal’s healthcare needs, providing skilled care in various settings, and contributing to the backbone of the nursing workforce, leading to registration as a registered nurse [8]. This bifurcated system reflects the diverse educational trajectories available to nursing students in Nepal, shaping their professional development and influencing their contributions to healthcare.

Irrespective of the qualification level, both pathways mandate supervised clinical experience within hospital settings as a fundamental and irreplaceable component of nursing education. Clinical practice constitutes 60% of the nursing curriculum, which is over 4000 h of clinical placement [9], allowing students to acquire and hone the essential skills necessary for safe and competent practice across various wards and hospital departments [10]. Crucial to this process is clinical supervision by appropriately qualified and experienced registered nurses, ensuring students attain the requisite clinical competencies outlined in the curriculum [11]. However, this supervisory role is becoming increasingly strained due to the escalating number of nursing students juxtaposed with a dwindling nursing workforce [12, 13]. The remaining staff are often burdened with overseeing a disproportionate number of students, which compromises the quality of supervision and impedes a conducive clinical learning environment [14, 15].

Existing literature has illuminated a range of obstacles that hinder effective clinical learning, challenges that are particularly pronounced in developing countries like Nepal [15, 16] but are also evident in high-income nations [17, 18]. These obstacles encompass factors such as insufficient teaching resources, communication barriers within healthcare teams and with patients, the cultural nuances and complexities of care, the dynamics of the supervisory relationship between students and preceptors, and resource limitations within healthcare organizations [19, 20]. Nepal, like many resource-limited countries, is grappling with a critical nursing shortage. The current nurse-to-population ratio of 3.4 per 1000 people [21], translating to fewer than 115,900 registered nurses serving a population of over 30 million, underscores the severity of this crisis [22]. This shortage is largely attributed to the phenomenon of “brain drain,” which refers to the migration of highly skilled individuals, including healthcare professionals, from their country of origin to other countries, often seeking better economic opportunities and working conditions [23]. This outward migration is particularly pronounced in Nepal, which experiences one of the highest rates of healthcare professional migration in the region [24]. Consequently, over a third of its nurses have sought licensure to practice abroad, primarily in high-income countries like the United States, the United Kingdom (UK), and Australia [22].

The ramifications of this brain drain have been extensively discussed in terms of patient safety [25] and the widening health inequities between the Global North and South [26], contributing to a pressing health workforce crisis in lower- and middle-income countries (LMICs) [27]. This global crisis is acutely felt in Nepal, where both authors, drawing from their extensive experience in clinical nursing education within teaching hospitals, have witnessed firsthand the myriad challenges students encounter in receiving adequate support during their clinical placements. While environmental [20, 28], economic [29, 30], social, and cultural conditions [31, 32] influencing nursing students’ needs have been well-documented, there remains a significant gap in the literature regarding the effects of staffing shortages on the clinical learning experience and its subsequent impact on nursing education. Thus, this study seeks to address this critical knowledge gap by investigating the central question: How does the staffing shortage affect the clinical learning environment for undergraduate nursing students during their clinical placement?

## Methods

### Study design

This study employed a qualitative research approach using both exploratory [33] and descriptive [34] designs to investigate nursing students’ lived experiences and perceptions regarding the support they received from nursing staff within the clinical learning environment. This approach enabled an in-depth understanding of the complex and subjective nature of students’ experiences, providing rich, contextualized data to illuminate the challenges and opportunities they encountered in the clinical setting. This study adhered to the Consolidated criteria for reporting qualitative research (COREQ) guidelines for reporting qualitative study [35].

### Sample and setting

Participants for this study were purposefully recruited from two nursing tertiary institutions located in Bharatpur, an urban center within the Chitwan district in Nepal. These institutions are recognized as the primary providers of tertiary education in the region. The strategic selection of these two sites was based on their substantial size and their distinction of having the largest cohorts of undergraduate nursing students.

To ensure that participants possessed a depth of clinical experience relevant to the research focus, inclusion criteria were established. Nursing students were eligible for participation if they met the following conditions: (1) they were enrolled in either the final or fourth year of their undergraduate program and (2) they had successfully completed a minimum of 75% of their required clinical placements. This approach aimed to capture the perspectives of students who had substantial exposure to the clinical learning environment during their clinical placement and could offer nuanced insights into the impact of staffing shortages on their educational experiences.

### Data collection

Data was collected through a series of focus group discussions (FGDs), adhering to the COREQ guidelines for reporting. Three FGDs were conducted, each comprising six final-year nursing students (*n* = 18). To foster a comfortable and conducive environment for discussion, the FGDs were held at the participants’ respective nursing schools at times convenient for them. Each group participated in a distinct FGD session that lasted approximately 120 min. The first two discussions took place in June 2024, with the third one in July 2024.

Recognizing the potential influence of pre-existing relationships on group dynamics, the composition of each FGD was carefully considered. FGD 1 consisted of students who had trained together consistently throughout their program, fostering a high degree of familiarity and shared experience within the group. In contrast, FGD 2 included students from different cohorts who had only interacted occasionally during their clinical rotations, while FGD 3 was composed of students who had never trained together, representing a group with minimal pre-existing relationships. This strategic variation in group composition aimed to capture a spectrum of perspectives and experiences, allowing for an exploration of how familiarity might influence the discussion and the insights generated.

A semi-structured interview guide was used to facilitate an in-depth exploration of the research topic (see Table 1). Participants were given the flexibility to respond in either English or Nepali, recognizing that while English is the language of instruction, expressing nuanced experiences in their native language might be more comfortable. All participants actively contributed their insights throughout the discussions, and the researchers employed probing and follow-up questions to elicit detailed information. All FGDs were audio-recorded with the explicit consent of the participants.

**Table 1:** Interview questions

Question Number	Focus Group Discussion Questions
1	Can you describe your typical day during a clinical placement?
2	How would you characterize the level of supervision and guidance you receive from nursing staff during your clinical placements?
3	In what ways has the nursing shortage affected your ability to learn and practice essential clinical skills?
4	Can you share any experiences where you felt your learning was compromised or hindered due to the lack of available nursing staff?
5	How has the nursing shortage affected your interactions and relationships with other healthcare professionals, such as doctors and allied health staff?
6	Have you found yourself seeking alternative sources of knowledge or support due to the limited availability of nursing staff? If so, can you describe these sources and experiences?
7	How has the nursing shortage affected your overall confidence and sense of preparedness for professional nursing practice?
8	What recommendations would you make to improve the clinical learning environment for nursing students in the context of the nursing shortage?

#### Data analysis

A thematic analysis approach was employed to analyze the qualitative data collected from the focus group discussions. This process, guided by the six-step framework outlined by Braun & Clarke [36], ensured a systematic and comprehensive interpretation of the data. To maintain consistency and minimize potential bias, all focus groups were moderated by the principal investigator (AG), a nursing academic with extensive experience in qualitative research and clinical education. Initially, the recorded FGDs were transcribed and, where necessary, translated verbatim by the principal investigator (AG). To enhance accuracy and mitigate potential bias, all translated scripts were cross-checked by a second researcher (MSN) proficient in both Nepali and English. The research team then engaged in multiple readings of the transcripts to gain a deep familiarity with the data. Subsequently, meaningful units of text were extracted from the transcripts, representing distinct ideas or experiences conveyed by the participants. These meaning units were then condensed to capture their essence while preserving their core meaning. Codes were assigned to these condensed meaning units, and a comprehensive codebook was developed to categorize and organize the codes into meaningful groups based on shared conceptual similarities. The study aimed for code and thematic saturation, as opposed to data saturation [37].

To ensure rigor and consensus in the thematic analysis, a collaborative approach was adopted. The research team, comprised of nursing academics with extensive experience in qualitative research and clinical education, engaged in regular meetings to discuss the coding process, emerging categories, and potential themes. Initial coding was conducted independently by two researchers (AG and MSN), and the codebook was iteratively refined through a process of comparison and discussion. Any discrepancies in coding or interpretation were resolved through consensus meetings, where the team reviewed the relevant data extracts and negotiated a shared understanding. This iterative process of independent coding, comparison, and discussion continued until the research team reached a consensus on the final set of themes and sub-themes.

Through this rigorous process, the initial codes coalesced into overarching themes that captured the salient patterns and insights within the data. In the final phase, a detailed narrative was constructed to present the thematic analysis findings, incorporating illustrative quotes from the participants to provide rich, contextualized evidence. To further enhance the trustworthiness of the analysis, peer debriefing was conducted, wherein an external expert in qualitative research reviewed the transcripts, methodology, and findings. This process allowed for critical feedback and identifying potential biases or inconsistencies. The research team revisited the transcripts and audio recordings as needed to ensure the final report accurately reflected the participants’ voices. The comprehensive data analysis process is summarized in Table 2.

**Table 2:** Data analysis process

Exemplar Meaning Unit	Code	Category	Theme
“…constantly treading water, just trying to keep our heads above the surface.” (*P12 Fgd 2)*	Overwhelmed, struggling to keep up	Lack of support and guidance	Compromised Learning: The Cascading Effects of Staff Shortages
“The wards are overflowing, and the nurses are stretched thin…we feel like a burden.” (*P1 Fgd 1)*	Feeling like a burden, hesitant to ask	Impact of nurse workload on student learning	Compromised Learning: The Cascading Effects of Staff Shortages
“With the constant chaos and lack of supervision, it feels like we’re just surviving, not thriving.” (*P18 Fgd 3)*	Survival mode, unpreparedness	Concerns about future competence	Compromised Learning: The Cascading Effects of Staff Shortages
“…a patient’s ventilator alarm started blaring. I froze… My supervisor was nowhere to be found.” *(P7 Fgd 2)*	Lack of supervision, feeling helpless	Isolation and lack of support	Abandoned in the Field: Isolation and the Erosion of Student Confidence
“We’re told that clinical placements are where we truly become nurses. But sometimes, it feels more like we’re just ghosts, silently observing from the sidelines.” *(P11 Fgd 2)*	Feeling invisible, underutilized	Missed learning opportunities	Abandoned in the Field: Isolation and the Erosion of Student Confidence
“Each time I hold back from asking a question… a little piece of my confidence chips away.” *(P15 Fgd 3)*	Erosion of confidence, self-doubt	Psychological impact of lack of support	Abandoned in the Field: Isolation and the Erosion of Student Confidence
“…I mustered up the courage to approach the attending physician during rounds. He patiently explained…” *(P3 Fgd 1)*	Seeking alternative knowledge sources, Proactive learning	Resourcefulness and adaptability	Forging Ahead: Unintended Resilience in the Face of Adversity
“…I decided to ask the respiratory therapist… She not only demonstrated the technique but also explained the physiological principles behind it.” *(P10 Fgd 2)*	Collaboration with allied health, Invaluable learning	Seeking expertise from other professionals	Forging Ahead: Unintended Resilience in the Face of Adversity
“When I’m unsure about something, I often turn to the patients themselves… learning can happen anywhere, even in the most unexpected places.” *(P16 Fgd3)*	Patient-centered care, Unexpected learning	Alternative sources of knowledge	Forging Ahead: Unintended Resilience in the Face of Adversity
“…I encountered a rare post-operative complication… I cautiously consulted an AI… It generated a list of possible diagnoses and suggested interventions” *(P4 Fgd 1)*	AI as a resource, Supplementing learning	Utilizing technology to bridge knowledge gaps	Digital Mentors: AI as a Lifeline in Overburdened Clinical Settings
“AI has become my go-to study buddy… It’s like having a 24/7 library at my fingertips.” *(P9 Fgd 2)*	AI for self-directed learning, Access to information	AI as a supplementary learning tool	Digital Mentors: AI as a Lifeline in Overburdened Clinical Settings
“I know AI can’t replace human interaction… But in a system stretched to its limits, AI has become an invaluable tool for self-directed learning.” *(P15 Fgd 3)*	AI limitations, Empowerment through technology	AI as a support system in understaffed settings	Digital Mentors: AI as a Lifeline in Overburdened Clinical Settings

### Rigor and reflexivity

To ensure the trustworthiness of the qualitative data and subsequent analysis, this study adhered to established criteria for rigor, encompassing credibility, dependability, confirmability, and transferability [38]. Credibility was established through member checking during data collection. This involved actively seeking confirmation from participants regarding the accuracy of interpretations and addressing any areas of misunderstanding or omission.

Member checking was conducted in two phases. First, during each focus group discussion, the moderator (AG) summarized key points and emerging interpretations to allow participants to confirm or challenge their understanding. This ongoing member checking during data collection ensured that the researcher’s interpretations were aligned with the participants’ intended meanings. Second, after the initial thematic analysis, a summary of the key themes and supporting quotes was compiled and sent to all 18 participants via email. They were invited to review the summary and provide feedback on the accuracy and resonance of the themes with their experiences. Participants were given two weeks to respond, and any feedback received was carefully considered and incorporated into the final analysis. Additionally, peer debriefing was employed, wherein an independent expert in qualitative research reviewed the transcripts, methodology, and emergent themes, offering critical feedback and ensuring the fidelity of the research process.

Dependability was achieved through the development of the data collection instrument based on a comprehensive literature review and the authors’ extensive clinical teaching experience, further strengthened by expert review from specialists in nursing education and qualitative methodology. Confirmability was ensured through transparent and detailed documentation of the analytical process. This included descriptions of how data were summarized, meaning units extracted, condensed, coded, and ultimately synthesized into categories and themes.

To enhance the confirmability and trustworthiness of the findings, reflexivity was addressed through several strategies. The research team, comprised of nursing academics with extensive clinical teaching backgrounds, engaged in ongoing reflection throughout the study. Recognizing that our own experiences as educators and clinicians might influence our interpretations, we maintained reflexive journals to document our assumptions, preconceptions, and potential biases. These journals served as a tool for critical self-reflection, allowing us to examine how our perspectives might shape the research process, from data collection to analysis and interpretation. Furthermore, regular team meetings provided a platform for open dialogue and reflexive discussions, where we challenged each other’s interpretations and ensured that the emerging themes were grounded in the data rather than our own preconceived notions.

Finally, transferability, which pertains to the potential applicability of the findings to other contexts, was supported through detailed descriptions of the study participants and an account of the research process. This comprehensive documentation allows readers to assess the relevance and transferability of the findings to their own settings and situations.

### Ethical consideration

Ethical clearance was obtained from the Nepal Health Research Council (approval number – 427/2024) and the institutional review boards of Chitwan Medial College and B.P. Koirala Memorial Cancer Hospital. Written voluntary informed consent was obtained from each participant, who was assured of their right to withdraw from the study at any time.

## Results

### Participants characteristics

Eighteen female nursing students aged 22 to 26 years participated in this study. All were recruited from two tertiary institutions in Bharatpur, Chitwan, Nepal. Eleven participants had enrolled in the Bachelor in Nursing program after completing their high school education, while seven had previously obtained a Proficiency Certificate Level (PCL) in Nursing. Eleven participants identified nursing as their first-choice career path, suggesting a degree of intentionality in their pursuit of this profession. The sample’s exclusively female composition reflects the prevailing gender dynamics in Nepal’s nursing landscape, where nursing is predominantly perceived as a female-dominated field. This observation is further substantiated by a report from the International Labor Organization indicating that the nursing workforce in Nepal is entirely female [24]. Table 3 provides a detailed overview of the participants’ socio-demographic characteristics.

**Table 3:** Participants’ socio-demographic characteristics

Participant	Gender	Previous Education	Age	Nursing as First Choice
P1	Female	High School	22	Yes
P2	Female	High School	22	Yes
P3	Female	High School	25	No
P4	Female	High School	24	Yes
P5	Female	High School	22	No
P6	Female	High School	26	Yes
P7	Female	High School	22	Yes
P8	Female	High School	23	No
P9	Female	High School	26	No
P10	Female	High School	24	No
P11	Female	High School	24	Yes
P12	Female	PCL Nursing Course	24	Yes
P13	Female	PCL Nursing Course	25	Yes
P14	Female	PCL Nursing Course	24	Yes
P15	Female	PCL Nursing Course	25	Yes
P16	Female	PCL Nursing Course	25	No
P17	Female	PCL Nursing Course	25	No
P18	Female	PCL Nursing Course	25	Yes

### Findings

#### Theme 1: compromised learning: the cascading effects of staff shortages

In Nepal, the clinical learning environment within hospital settings is undergoing a profound transformation in the face of a critical nursing shortage. The scarcity of experienced nurses has ignited a chain reaction, leaving students grappling with compromised learning opportunities and an uncertain educational journey. Their voices echo the far-reaching consequences of this crisis, painting a vivid picture of a strained system struggling to nurture the next generation of healthcare providers.

It feels like we’re constantly treading water, just trying to keep our heads above the surface. With so few nurses on the floor, there’s simply no time for them to teach us. We’re often left to observe from a distance, missing out on those crucial hands-on experiences that solidify our understanding. (P12 Fgd 2)

This quote illustrates the struggle and missed opportunities that characterize the compromised learning environment.

The wards are overflowing, and the nurses are stretched too thin. It’s heartbreaking to see them rushing from patient to patient, barely able to catch their breath. In those moments, we feel like a burden, another task on their already overflowing plate. It makes it hard to ask questions or seek guidance, even though we desperately need it. (P1 Fgd 1)

This student’s reflection highlights the emotional toll of the nursing shortage on both students and nurses. The overwhelming workload and lack of time create barriers to effective teaching and mentorship.

We came here to learn and to grow into competent nurses. But with the constant chaos and lack of supervision, it feels like we’re just surviving, not thriving. We worry that we’re not getting the preparation we need to provide safe and effective care to our future patients. (P18 Fgd 3)

This quote encapsulates the overarching concern of compromised learning: the fear of being ill-prepared for professional practice.

### Theme 2: abandoned in the field: isolation and the erosion of student confidence

The nursing shortage in Nepal has not only compromised the technical aspects of clinical learning but also created a pervasive sense of isolation among students. This theme explores the emotional and psychological consequences of being left to navigate the complexities of clinical practice with minimal guidance and support.

I remember one day in the ICU, a patient’s ventilator alarm started beeping. I froze, unsure of what to do. I looked around for my supervisor, but she was nowhere to be found. I so desperately wanted to learn how to troubleshoot the machine and respond to the patient’s needs, but I didn’t dare interrupt the nurses who were already frantically trying to manage the situation. (P7 Fgd 2)

This student’s experience captures the feelings of abandonment and helplessness in an understaffed clinical environment. The lack of readily available supervision leaves students unprepared to handle critical situations.

We’re told that clinical placements are where we truly become nurses. But sometimes, it feels more like we’re just ghosts, silently observing from the sidelines. I’ve been longing to practice inserting intravenous (IV) lines, but I hesitate to ask. The nurses are so busy, and I don’t want to be seen as another demand on their time. (P11 Fgd 2)

This quote highlights the disconnect between the idealized vision of clinical placements and the reality experienced by students in an understaffed setting.

Each time I hold back from asking a question or seeking help, a little piece of my confidence chips away. I worry that I’ll never be truly ready for the real world of nursing. It’s a constant battle between wanting to learn and fearing that I’ll be seen as incompetent or demanding. (P15 Fgd 3)

This reflection reveals the insidious impact of isolation and lack of support on students’ self-confidence. The fear of judgment and the perceived need to cope independently create a cycle of self-doubt, hindering their professional development and affecting their long-term career aspirations.

### Theme 3: forging ahead: unintended resilience in the face of adversity

While the nursing shortage in Nepal creates formidable challenges for student learning, it has also inadvertently fostered a remarkable sense of resilience and resourcefulness among aspiring nurses. In the absence of consistent mentorship and guidance, these students have carved their own paths, actively seeking knowledge from unexpected sources. Their stories highlight the missed opportunities for traditional mentorship but also showcase the unwavering determination of a generation eager to learn and serve.

One day, I was struggling to understand the intricacies of a patient’s complex medication regimen. My supervisor was swamped, so I mustered up the courage to approach the attending physician during rounds. He patiently explained the rationale behind each drug and even shared some valuable clinical pearls. It was a nerve-wracking experience, but it taught me the importance of proactive learning and seeking out knowledge wherever I can find it. (P3 Fgd 1)

This student’s initiative to seek guidance from the attending physician exemplifies the resourcefulness born out of necessity.

I’ll never forget the time I was caring for a patient with a tracheostomy. I had so many questions about the suctioning procedure, but my supervisor was busy with other patients […] I decided to ask the respiratory therapist who was working with the patient. She not only demonstrated the technique but also explained the physiological principles behind it. It was an invaluable learning experience. (P10 Fgd 2)

This anecdote highlights the collaborative spirit that emerges when traditional mentorship structures are strained. The student recognized the respiratory therapist’s expertise and seized the opportunity to learn, demonstrating an openness to interprofessional collaboration and a commitment to acquiring knowledge from diverse sources.

When I’m unsure about something, I often turn to the patients themselves. They’re a wealth of knowledge about their own conditions and experiences. I once had a patient with diabetes who taught me so much about the challenges of managing the disease. It was a humbling reminder that learning can happen anywhere, even in the most unexpected places. (P16 Fgd3)

This student’s reflection highlights the importance of patient-centered care and the unique learning opportunities that arise from engaging with patients as partners in the learning process.

### Theme 4: digital mentors: Artificial Intelligence (AI) as a lifeline in overburdened clinical settings

In the face of overwhelming demands and limited human resources, a new ally has emerged for nursing students in Nepal: artificial intelligence. As traditional support systems buckle under the strain of the nursing shortage, AI is stepping in to fill the gaps, offering a lifeline of information and guidance. While there is no replacement for human connection and mentorship, AI is proving to be a transformative tool, enabling students to learn interactively and bridge the knowledge gaps that arise in overburdened clinical settings. Their experiences highlight the innovative ways in which technology is shaping the future of nursing education, and it raises important questions about the evolving role of AI in healthcare training.

During my surgical rotation, I encountered a rare post-operative complication. I couldn’t find much information in our textbooks, and the nurses were too busy to explain. I cautiously consulted an AI […]. It generated a list of possible diagnoses and suggested nursing interventions. It wasn’t a replacement for expert guidance, but it helped me feel less lost and gave me a starting point for further research and discussion with my supervisor. (P4 Fgd 1)

This student’s experience highlights the potential of AI to bridge knowledge gaps and empower students to take initiative in their learning.

I’ve always struggled with interpreting ECG (Electrocardiogram) readings. It’s one of those skills that requires a lot of practice and expert guidance. But with my supervisor constantly running from one crisis to the next, I rarely had the opportunity to ask for help. That’s when I discovered an ECG interpretation app. The interactive modules and simulations brought the complex rhythms to life, allowing me to visualize and understand them in a way that textbooks couldn’t. It was like having a personal tutor available at my fingertips, even in the middle of the night! (P14 Fgd3)

The interactive nature of AI-powered tools can enhance understanding and skill development, especially in areas where direct supervision might be limited.

AI has become my go-to study buddy. It provides me with concise, evidence-based information and even suggests relevant articles and videos. It’s like having a 24/7 library at my fingertips, allowing me to learn at my own pace and fill in the gaps in my knowledge. (P9 Fgd 2)

In a resource-constrained environment, AI can provide on-demand access to information and support, empowering students to take control of their learning journey.

I know AI can’t replace human interaction, and I still crave the guidance of experienced nurses. But in a system stretched to its limits, AI has become an invaluable tool for self-directed learning. It’s empowering to know that I can access information and support whenever I need it, even when my supervisors are overwhelmed. (P15 Fgd 3)

This quote encapsulates the balanced perspective on AI as a valuable supplement to, but not a replacement for, human mentorship.

## Discussion

The nursing shortage in Nepal, as illuminated in this study, profoundly influences the clinical learning environment, significantly impacting undergraduate nursing students’ acquisition of essential clinical skills. Overburdened clinical staff grappling with patient care demands amidst staffing constraints inadvertently triggers a cascade of missed learning opportunities for students. This phenomenon resonates with a growing body of evidence that underscores the detrimental effects of nursing shortages on clinical education globally [12]. Research consistently demonstrates that inadequate staffing levels negatively impact students’ ability to gain hands-on experience, receive timely feedback, and develop critical thinking skills, thereby widening the existing theory-practice gap [39, 40]. In the context of Nepal, where the nursing shortage is particularly acute [22, 41], these challenges are magnified, hindering the development of confidence and competence required for safe and effective patient care. This compromised learning environment affects students’ immediate skill acquisition and raises serious concerns about their preparedness for professional practice. The long-term implications are significant, perpetuating a cycle of under-preparedness with a burgeoning question of nursing graduates’ “work readiness” [42] and contributing to the ongoing shortage through increased attrition rates [11] – a pressing global issue within the nursing profession.

Furthermore, the study’s findings, viewed through the lens of Bandura’s social cognitive theory [43], reveal that the impact of the nursing shortage extends beyond the student-supervisor relationship. The theory posits that learning occurs in a social context through observation, modeling, and reinforcement [43]. In an understaffed environment, overwhelmed clinical staff are often stretched to their limits. This can inadvertently lead them to model behaviors of stress and frustration, creating a learning environment where students feel like a “burden” rather than valued learners. This perceived lack of support leads to hesitancy in seeking guidance or asking questions, further impeding the learning process and hindering the development of self-efficacy among students. The ripple effects of the nursing shortage thus extend beyond immediate skill acquisition, shaping students’ professional identity formation and potentially influencing their long-term commitment to the nursing profession [44]. While specific to Nepal, this study’s findings resonate with challenges observed in other resource-constrained settings, suggesting a global need to address the systemic issues underlying the nursing shortage and its impact on clinical education [44].

The substantial clinical hour requirement in Nepal’s undergraduate nursing programs (over 4000 h) raises pertinent questions about the optimal balance between theoretical and practical training [9]. While clinical immersion is undeniably crucial, the mandated intensity of clinical placements in Nepal, coupled with the nursing shortage, creates a significant strain on the supervisory capacity of qualified nurses [10]. This echoes global debates surrounding the mandated clinical hours in nursing education [45]. A contrasting example is the Australian model, requiring 800 h of clinical practice [46], compared to other countries like New Zealand, requiring 1,100 h [47], and the UK, with 2,300 h [48], over a three-year period. This disparity underscores the ongoing debate surrounding the optimal balance between clinical immersion and alternative pedagogical approaches.

Incorporating simulated clinical learning experiences is a valuable strategy to alleviate the pressure on overburdened clinical settings [49] in Nepal while addressing concerns about reduced clinical hours. Simulation offers a safe and controlled environment for students to practice skills, develop critical thinking, and gain confidence before encountering real-life clinical scenarios [50]. High-fidelity simulations, using advanced mannequins and virtual reality technology, can replicate complex clinical situations, allowing students to hone their clinical judgment and decision-making skills without the risk of harming real patients [51]. Furthermore, simulation can be tailored to address specific learning needs and provide opportunities for deliberate practice and feedback [52]. For instance, students can practice responding to emergency situations, managing complex medication regimens, or communicating with challenging patients in a simulated environment. This allows them to make mistakes, learn from them, and refine their skills before applying them in real-world settings.

However, it is essential to acknowledge that simulation does not serve as a comprehensive solution. While it can be a powerful tool to supplement clinical learning, it cannot entirely replace the authentic experience of interacting with real patients and navigating the complexities of the healthcare environment [53]. The ideal approach likely lies in a judicious blend of simulation, traditional clinical placements, and innovative teaching methods, tailored to the specific needs of students and the resources available. By critically examining the mandated clinical hours and exploring the potential of simulated learning, Nepal can contribute to the global dialogue on optimizing nursing education. A more balanced and flexible approach, incorporating diverse learning methods, could enhance the quality of nursing education, alleviate the burden on clinical settings, and ultimately contribute to a more competent and confident nursing workforce.

The scarcity of nursing staff has not only compromised the technical aspects of clinical learning but has also engendered a palpable sense of isolation and abandonment among nursing students. Students’ poignant descriptions of feeling like “ghosts, silently observing from the sidelines” paint a stark picture of a clinical learning environment where their presence feels undervalued and their learning needs unmet. This isolation, exacerbated by the lack of adequate supervision and mentorship, has a profound impact on students’ confidence and sense of belonging, hindering the social learning processes crucial for their development as nurses [54]. Nonetheless, the importance of mentorship in clinical education cannot be overstated. Mentors serve as role models, provide technical guidance and emotional support, facilitate professional socialization, and promote self-efficacy among students [55]. Previous research has consistently demonstrated that strong mentorship programs can significantly enhance clinical learning experience, fostering a sense of belonging and empowerment [56, 57]. In the context of a nursing shortage, where students feel particularly vulnerable and unsupported, the absence of mentorship can be acutely felt. This sense of invisibility and lack of guidance make students feel disconnected from nursing during the very formative years of their learning [56, 58]. This disconnection may be further exacerbated by the prevailing gender dynamics within the profession.

The exclusive female participation in this study reflects the gendered nature of nursing in Nepal, where it is predominantly perceived as a female profession [24]. This societal construct, coupled with the hierarchical structures and male dominance often found in healthcare settings [59], can contribute to the feelings of “invisibility” reported by the participants. Female nursing students face additional challenges in asserting their needs and seeking support within this environment [60], hindering their ability to actively engage in the learning process and advocate for their educational needs. Female students, socialized to be respectful and avoid questioning superiors, hesitate to seek clarification or express concerns, even when faced with compromised learning opportunities [61]. This can lead to a sense of isolation and hinder their ability to actively engage in the learning process. Moreover, these challenges are mirrored in the experiences of nurses working in other resource-constrained settings [44]. The experience of professional isolation and “enforced loneliness” highlighted in previous research [62, 63] resonates with the feelings of abandonment expressed by Nepalese nursing students. This suggests that the impact of the nursing shortage on the CLE is not only a systemic challenge but also one that intersects with cultural and contextual factors, necessitating tailored solutions. The pervasive sense of isolation experienced by nursing students in Nepal highlights a critical gap in the clinical learning environment, perpetuating a cycle of missed learning opportunities and contributing to feelings of inadequacy and self-doubt. While students are demonstrating resilience in seeking alternative avenues for learning, we must address the root causes of this isolation to ensure the development of a confident and competent nursing workforce.

Paradoxically, the nursing shortage in Nepal, while presenting a formidable challenge, has also inadvertently fostered a remarkable sense of resilience and resourcefulness among students, a phenomenon that can be understood through the lens of resilience theory [64, 65]. Faced with a dearth of direct mentorship and guidance, they have demonstrated a remarkable ability to adapt and forge their own learning pathways in response to the isolation and lack of mentorship described earlier. Seeking knowledge from alternative sources, such as physicians, allied health professionals, and even patients themselves [66], speaks to their unwavering determination to succeed. However, this newfound resilience, while commendable, should not be misconstrued as a sustainable solution to the nursing shortage’s impact on clinical education. It is crucial to recognize that these students are essentially compensating for a systemic failure, and their proactive efforts should not absolve the healthcare system of its responsibility to provide adequate support and mentorship.

If left unaddressed, this reliance on self-directed learning could create a two-tiered system where students with greater initiative, access to resources, and higher socioeconomic status thrive. In contrast, those who struggle to navigate the complexities of an understaffed environment are left behind, further perpetuating existing inequities in education and healthcare. Furthermore, the reliance on alternative sources of knowledge raises concerns about the potential for misinformation and inconsistent guidance, potentially impacting students’ ability to develop evidence-based practice skills. Although physicians and allied health professionals possess valuable expertise, they may not always be equipped to provide the nuanced, nursing-specific instruction that students require. Likewise, while offering unique insights into their lived experiences, patients cannot replace the evidence-based rationale, structured mentorship, and feedback that clinical instructors provide. Self-directed learning, though valuable for fostering autonomy, problem-solving skills, and critical thinking, should not replace structured mentorship and guidance. If this trend of self-directed learning persists without intervention, it could lead to a generation of new nurses who, while resilient and adaptable, may lack the depth and breadth of knowledge that comes from comprehensive, supervised clinical education. This could have long-term implications for the quality of patient care, potentially undermining the very foundation of the healthcare system.

The emergence of AI as a “digital mentor” in the Nepalese clinical learning environment speaks volumes about the innovative spirit of nursing students and the evolving landscape of healthcare education. The nursing shortage has created a challenging learning environment marked by isolation, lack of supervision, and compromised learning opportunities. Faced with these limitations, students have embraced AI-powered tools, leveraging their interactive modules, simulations, and on-demand information to bridge knowledge gaps and supplement their learning. This resourceful adaptation aligns with the principles of the Technological Pedagogical Content Knowledge (TPACK) framework, where technology is integrated to enhance pedagogical practices and content knowledge [67, 68]. For instance, one student shared how an AI-powered ECG interpretation app helped them visualize and understand complex cardiac rhythms, providing a valuable learning experience that was otherwise inaccessible due to the limited availability of their supervisor. This resourceful adaptation underscores the potential of AI to revolutionize nursing education, particularly in resource-constrained settings [69]. AI’s ability to offer interactive modules, simulations, and on-demand information provides students with a degree of autonomy and flexibility in their learning [70], mitigating the challenges posed by overburdened clinical staff. Recent studies support the positive impact of AI in nursing education, with students expressing favorable views on the use of generative AI tools for their personalized and interactive learning experiences [71, 72].

However, while AI offers promising opportunities, it is important to approach its integration into nursing education with a critical eye, acknowledging both its potential and limitations. While AI can enhance access to information and provide tailored learning experiences, it cannot fully replicate the guidance, emotional support, and role modeling provided by experienced nursing mentors [73]. Human mentors possess empathy and contextual awareness to address students’ individual needs and anxieties, fostering a sense of belonging and professional identity formation that AI cannot replicate. Furthermore, studies have shown that AI models can exhibit shortcomings in clinical decision-making, including indecisiveness and a tendency to over-recommend diagnostic tests [74, 75]. This raises ethical concerns about the potential for AI to perpetuate biases or lead to errors in judgment if not used judiciously. For instance, if an AI model is trained on biased data, it may inadvertently reinforce existing healthcare disparities or lead to inaccurate diagnoses for underrepresented populations [76]. Moreover, the “black box” nature of some AI algorithms can make it difficult to understand the reasoning behind their recommendations [77], potentially hindering students’ ability to develop critical thinking and clinical reasoning skills.

To harness the benefits of AI while mitigating its risks, a balanced and thoughtful approach to its integration into nursing education is crucial. AI should be viewed as a powerful supplement, a scalpel rather than a panacea, enriching traditional learning methods and human mentorship rather than replacing them [78]. Nursing programs should prioritize the development of students’ critical thinking and ethical reasoning skills, ensuring they can evaluate AI-generated information critically, identify potential biases, and make informed decisions in complex clinical situations. This includes fostering a mindset of “informed skepticism” towards AI, encouraging students to question its outputs and seek clarification from human mentors when needed. Ultimately, the successful integration of AI into nursing education requires a synergistic approach that combines the strengths of both human and artificial intelligence. By embracing AI’s potential while remaining vigilant about its limitations, we can create a learning environment that empowers students with knowledge, skills, and the critical thinking necessary to navigate the complexities of 21st-century healthcare.

### Limitations and future research

While this study offers valuable insights into the impact of the nursing shortage on clinical education in Nepal, it is essential to acknowledge the boundaries of its scope. The qualitative design, while allowing for a rich exploration of students’ experiences, inherently limits the generalizability of the findings to other contexts. The sample, drawn from two institutions in Bharatpur, Nepal, a relatively urbanized region, may not fully represent the diverse realities of nursing students in rural areas or those from marginalized socioeconomic backgrounds. Furthermore, by focusing on final-year students, the study excludes the perspectives of younger cohorts who might encounter different challenges earlier in their training. For instance, junior students, with their limited clinical experience, might be even more vulnerable to feelings of isolation and may struggle to compensate for the lack of supervision through self-directed learning. Additionally, the study’s focus on undergraduate nursing students leaves a gap in understanding the experiences of postgraduate learners and practicing nurses. It is plausible that the nursing shortage has a cascading effect throughout the profession, impacting the quality of mentorship available to postgraduate students and contributing to burnout and job dissatisfaction among practicing nurses. Future research exploring these perspectives would provide a more holistic understanding of the crisis. Hence, it is important to approach the findings of this study with a nuanced perspective, recognizing that they may not be fully transferable to other settings or populations. Nevertheless, this research provides a valuable starting point for understanding the challenges faced by nursing students in Nepal and highlights the urgent need for systemic change to address the nursing shortage and its far-reaching consequences.

Therefore, future research should expand upon these findings by employing larger and more diverse samples, encompassing students from various regions and institutions across Nepal and, ideally, including students from other countries experiencing similar nurse shortages. This broader perspective would allow for comparative analyses and a deeper understanding of the cultural and contextual factors influencing student experiences. Additionally, longitudinal studies could shed light on the long-term consequences of compromised clinical learning environments on nursing graduates’ professional trajectories, job satisfaction, work readiness upon graduation, and patient care outcomes. Finally, future studies should explore the potential and limitations of AI in nursing education, investigating its effectiveness for specific learning objectives, ethical implications, and its role in fostering critical thinking and clinical reasoning skills. Such research can contribute to developing evidence-based strategies that support and empower nursing students in resource-constrained settings, ultimately strengthening the nursing workforce and enhancing the quality of patient care in Nepal and beyond.

## Conclusion and recommendations

This study illuminates a stark reality: the nursing shortage in Nepal is not merely a staffing issue; it is a crisis that affects the future of healthcare. The compromised learning environment, the isolation felt by students, and their reliance on AI and alternative learning sources signal a system under strain. While the resilience and adaptability of these aspiring nurses are commendable, they cannot be a substitute for systemic reform. A healthcare system where every nursing student feels supported, guided, and empowered to reach their full potential. A system where mentorship is a cornerstone of clinical education. A system where technology complements, not compensates for, the essential human element in healthcare training. This is the vision we must strive for. To achieve this vision, a paradigm shift is required—a shift that moves beyond superficial measures and addresses the root causes of the nursing shortage. This requires a multi-pronged approach and a concerted effort from policymakers, educators, and healthcare leaders to reimagine nursing education and workforce development in Nepal.

### Recommendations

1. **Prioritize Investment in the Nursing Workforce**: The Nepalese government and healthcare leaders must make a concerted effort to attract and retain nurses by offering competitive salaries, improving working conditions, and providing opportunities for professional development. Furthermore, policies aimed at addressing the root causes of the nursing shortage, such as the “brain drain” phenomenon, must be implemented. This could include incentivizing nurses to stay in Nepal through loan forgiveness programs, scholarship opportunities, or targeted recruitment campaigns in rural areas.
2. **Cultivate a Culture of Mentorship and Support**: Nursing programs and clinical settings must prioritize the creation of a supportive learning environment, even amidst staffing challenges. This includes establishing formal mentorship programs with a designated clinical supervisor nominated for all clinical settings across healthcare organizations. Facilitating peer support networks and encouraging open communication and collaboration between students and clinical staff can also foster a sense of belonging and empowerment.
3. **Embrace Technology Strategically**: Integrate AI and other technologies into nursing curricula strategically, providing training on their ethical and effective use. Explore innovative pedagogical approaches that leverage technology to enhance student engagement and self-directed learning but always emphasize the irreplaceable value of human interaction and mentorship.

The challenges facing nursing education in Nepal are complex and intertwined with the broader healthcare crisis. However, the insights generated by this study, in conjunction with other research and stakeholder engagement, can inform the development of effective strategies to strengthen the nursing workforce and pave the way for a brighter future for nurses and their patients. Nepal’s healthcare future hinges on our collective commitment to nurturing and empowering the next generation of nurses.

## Supplementary Information

Supplementary Material 1.

## Data Availability

The data supporting this study’s findings are available on request from the corresponding author. However, the data is not publicly available due to privacy or ethical restrictions.
